# ‘Essential’ arterial hypertension: time for a paradigm change

**DOI:** 10.1097/HJH.0000000000003767

**Published:** 2024-05-08

**Authors:** Gian Paolo Rossi, Domenico Bagordo, Federico B. Rossi, Giovanni Pintus, Giacomo Rossitto, Teresa M. Seccia

**Affiliations:** a Internal & Emergency Medicine; b Specialized Hypertension Center; c ARHYVAB, International PhD Program in Arterial Hypertension and Vascular Biology, Department of Medicine – DIMED and Department of Biomedical Sciences, University of Padova, Padova; d Department of Clinical, Internal, Anesthesiological and Cardiovascular Sciences, “Sapienza” University of Rome, Rome, Italy; e School of Cardiovascular & Metabolic Health, University of Glasgow, Glasgow, UK

**Keywords:** arterial hypertension, essential hypertension, primary aldosteronism, secondary hypertension

## Abstract

The exclusion of causes of hypertension is not systematically exploited in clinical practice. Therefore, essential hypertension is consistently presented as the most prevalent ‘cause’. The paradox of a condition with unknown causes being described as a common cause of hypertension translates into a diagnosis of essential hypertension in most patients, which precludes the detection of a curable cause of hypertension. The aim of this review is to investigate how the notion of essential hypertension has developed and whether scientific evidence still support the notion of its high prevalence by examining the most recent studies. These studies provided solid scientific evidence that, when systematically sought for, secondary hypertension is quite common and that secondary hypertension is highly prevalent. The increased awareness should lead to a systematic search for, with the goal of curing or achieving a better control of high blood pressure, and ultimately improving patients’ quality of life.

## INTRODUCTION

In 1933, Wilfred Trotter, one of the leading physicians and surgeons of the last century in the UK, wrote ‘The fundamental activity of medical science is to determine the ultimate causation of disease’ [1]. The cause(s) of many common diseases were, therefore, discovered following this recommendation and are now well known. Hence, after nine decades of mechanistic research, who would dare to challenge this statement by affirming that myocardial infarction and diabetes mellitus, just to mention two highly prevalent conditions, have no well defined cause? Probably no one. Yet, oddly enough, every day we face patients diagnosed with ‘essential’ hypertension, which by no means is a diagnosis, but rather the exclusion of multiple diagnoses that, most of the times, were not systematically ruled out. Notwithstanding this, the term ‘essential’ or primary hypertension still pervades the literature.

How and why have we reached this stage? The term ‘essential’ hypertension appeared for the first time in 1752: ‘we still admit the challenging existence of a type of arterial HT not precipitated by any known primary pathogenic event and traceable to no other preceding disease’ [2]. Along the same line, in 1930 Fishberg stated that ‘essential HT includes those cases of chronic HT, which neither clinically nor anatomically, can be demonstrated to have evolved from antecedent inflammatory disease of the kidneys or urinary obstruction’ [3]. One year after, Kaname Yoshimura, in his Senior Thesis at the University of Nebraska, described essential hypertension as ‘differentiated from the HT secondary to glomerular nephritis, urinary obstruction, certain forms of nephrosis, aortic insufficiency, arteriovenous aneurysm, hyperthyroidism, toxemia of pregnancy, HT of menopause, and other forms of transitory rise in blood pressure’ [4]. In those years, two of the most common secondary causes of hypertension other than chronic kidney disease, had not been identified and, therefore, these definitions came to no surprise. In fact, the works of Harry Goldblatt and Jerome Conn were yet to be published [5,6], and uncertainties even existed on whether pheochromocytoma could cause human hypertension [4]. The term ‘essential’ became synonymous with idiopathic, or primary, hypertension, for example, a form of arterial hypertension with no identifiable cause, which, furthermore, acquired a benign connotation.

In fact, physiologists started using the term ‘essential’ to underlie the fact that blood pressure (BP) was necessary to ensure perfusion of all the vital organs. Back then, the prevailing concept was that high BP values allowed a better perfusion of these organs and, thus, denoted a good state of health. Indeed, Paul Dudley White, one of the most influential Cardiologists of the past century and a founder of the American Heart Association, used to state that ‘HT may be an important compensatory mechanism, which should not be tampered with, even were it certain we could control it’ [7]. Thus, the adjective ‘essential’ applied to high BP acquired a ‘benign’ connotation, apart from ignorance of the underlying cause.

Even though the data of health insurance companies identified high BP as a prognosticator of poor outcomes, the view that essential hypertension was benign and associated with a good state of health persisted until publication of the Australian Study in Mild Hypertension in 1980 [8], followed by a number of other randomized clinical studies [9,10]. Altogether, these studies finally provided unequivocal evidence that antihypertensive treatment lowered cardiovascular deaths and events over placebo, which led to recognize hypertension as a correctable risk factor. Not surprisingly, for the reasons discussed below, essential hypertension started to be regarded as the most prevalent form of arterial hypertension and even to be described as the most common ‘cause’ of high BP, which is clearly a contradictory statement for a condition whose causes were, and still are, unknown.

### The prevalence of essential hypertension

Although the first editions of Harrison's Principle of Internal Medicine reported no figures of prevalence for different forms of hypertension, all current medical textbooks still describe essential hypertension as to account for 95–99% of all forms of human arterial hypertension. A rate of 5.8% for secondary hypertension, implying that over 94% were essential, made its first appearance starting from the 10th edition of the textbook, based on a study by Berglund *et al.*[11], which investigated a random sample of 689 middle-age (47 to 54 year old) Swedish white men in 1976. The study excluded young individuals, women, and patients with resistant hypertension, that is, the very subgroups wherein secondary hypertension is more common. At that time, the diagnostic work-up for secondary hypertension was rudimental, as one can easily imagine. Not surprisingly, but certainly questionably, the authors concluded that ‘in middle-aged men found to have HT at screening, extensive investigation aimed at detecting secondary HT are not necessary’.

From that timepoint on, this notion was taken for granted. In fact, these figures on the low prevalence of secondary hypertension could be traced along most medical literature, albeit with no further references and/or scientific basis. Despite the lack of solid evidence that these figures are correct, this notion was not even held worth of referencing and the proclamation that the vast majority of the cases of human arterial hypertension are ‘caused’ by essential hypertension spread through textbooks, expert statements [12] and even guidelines by major scientific societies.

In 2003, the ESH/ESC joint guidelines stated that ‘a specific cause of BP elevation can be identified in a minority (from less than 5 to 10%) of adult patients with HT’ [13]. Along the same line, the 2017 AHA/ACC guidelines affirmed that ‘a specific, remediable cause of HT can be identified in approximately 10% of adults with HT’ but stated that ‘if a cause can be correctly diagnosed and treated, patients with secondary HT can achieve a cure or experience a marked improvement in BP control, with reduction in cardiovascular disease risk’ [14]. Low figures appeared also in the ISH 2020 guidelines, which, however, acknowledged that ‘early diagnosis of secondary HT and the institution of appropriate targeted treatment have the potential to cure HT in some patients, or improve BP control/reduce the number of prescribed antihypertensive medications, in others’ [15]. Finally, the last ESH 2023 guidelines underlined that ‘secondary forms of HT account for only a small fraction of the overall HT prevalence, which is largely due to primary (essential) HT’, but honestly recognized that ‘their true prevalence is not precisely known, because available data may be confounded by the selection bias of the studies reported in the literature, the number of undiagnosed cases, and the varying definition of secondary forms of HT’ [16].

As of today, a careful search of the literature and available databases, including PubMed, Scopus and Web of Science, using the keywords ‘arterial HT’, ‘prevalence’, ‘essential’, ‘humans’, ‘rate’, and the Boolean operators ‘AND’, allowed retrieval of 91 clinical trials in PubMed, 126 articles in Scopus and 120 in Web-Of-Science. Unfortunately, most of the studies were observational and retrospective, and by no means met the quality criteria, listed in Table 1, required to provide scientifically solid evidence.

**TABLE 1:** Criteria that should be followed in studies of disease prevalence to provide scientifically solid evidence

• Definition of the population of interest
• Adequately large sample size
• No prior selection of patients
• Recruitment of a cohort that is representative of the general population of the hypertensive patients
• Consecutive recruitment of affected and unaffected individuals
• Unambiguous definition of the clinical phenotype
• Predefined diagnostic criteria
• Availability of a diagnostic test (or set of tests) validated against a ‘gold’ or ‘golden’ reference following the STARD recommendations [47]

A quick look at these criteria reveals that a major hurdle to determine the prevalence of essential hypertension, among all, is the lack of a precise definition of the phenotype of essential hypertension and of an accurate diagnostic test to unambiguously identify it. As discussed later, this test will likely never exist, because essential hypertension entails a variety of different conditions that are unlikely to be picked up by any single test. Therefore, the afore-mentioned figures on the low prevalence of secondary hypertension are not evidence-based and the high prevalence of primary (essential) hypertension is ‘the mysterious viability of the false’ as Sir George Pickering wrote, quoting Wilfried Trotter [17].

### Why essential hypertension is still over-diagnosed

Essential hypertension is commonly defined as a form of human hypertension where the underlying cause has not been identified, because if it were, the diagnosis would then be of ‘secondary’, not ‘essential’ hypertension. From the practical standpoint, this definition implies that diagnosing ‘essential’ hypertension requires that a thorough search for the causes of hypertension has been undertaken. Our everyday experience instead indicates that, under most circumstances, even in countries with a well developed national health system, this search is not done in clinical practice by doctors dealing with hypertensive patients.

There are several reasons underlying this scarce search and detection of secondary hypertension. The first is the mistaken belief that secondary forms of hypertension are exceptional, rather than being the rule, which means that the prior (pretest) probability of their detection is perceived as low. Therefore, in line with what reported in textbooks and taught in Medical Schools, practicing doctors are generally driven to believe that the quest for secondary hypertension, apart from being held as time-consuming and costly, is not rewarding. This was clearly shown by a retrospective survey of general practitioners in two European countries with a well developed healthcare system, as Germany and Italy, which showed that they estimated the prevalence of secondary hypertension to be 3% or less and ordered tests for searching it in less than 2% of their patients [18].

The second reason for neglecting the search of secondary hypertension involves the complexity of the diagnostic work-up, both in terms of planning of the tests that are required and recommended by guidelines, and of interpretation of their results. Accordingly, even the most enterprising and courageous doctors who decide to order the tests, face difficulties in interpreting their results, which explains why secondary hypertension is rarely detected. Possible solutions to this problem are simplified diagnostic algorithms, as proposed for primary aldosteronism and other conditions [19,20].

The third reason entails uncertainties on the outcome and worries on the consequences that might derive from diagnosing a secondary form of hypertension. For example, detecting primary aldosteronism in a patient seeking surgical cure implies the need for subtyping by means of adrenal vein sampling which, being a technically demanding and difficult-to-interpret procedure, remains confined to few centres worldwide [21]. Moreover, in some forms, for example pheochromocytoma-paraganglioma (PPGL), making the diagnosis implies genetic testing and, if germ-line mutations are found, the undertaking of demanding actions, such as the search for multiple tumours, extended work-up of first-degree family members, genetic counselling and so on [22], which, admittedly, are not easy undertakings for busy general practitioners.

**FIGURE 1:**
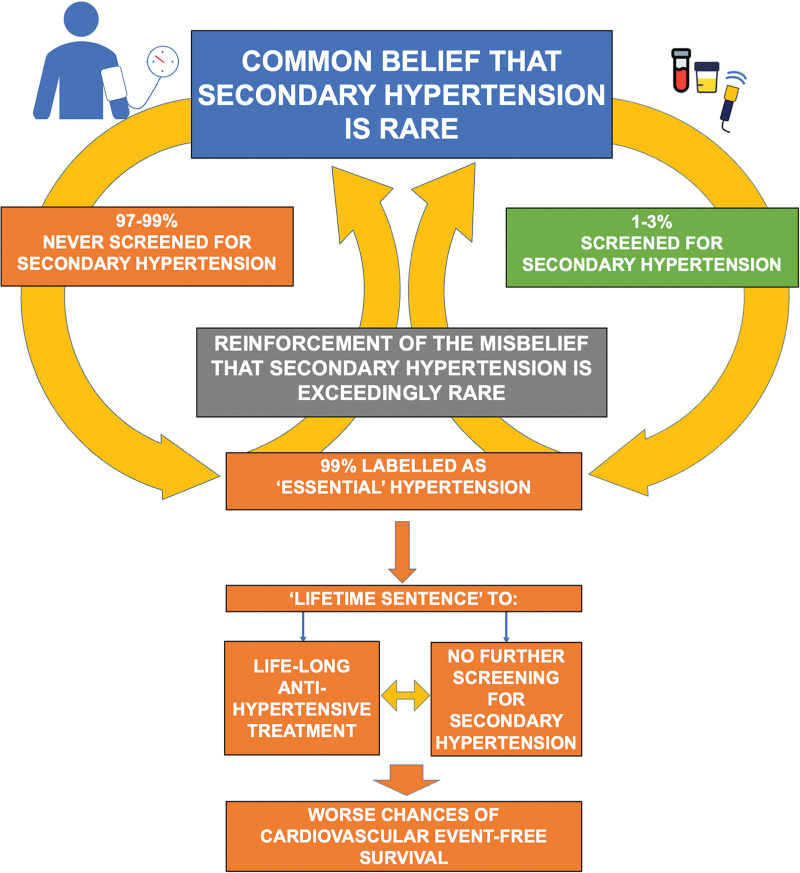
The cartoon illustrates the vicious circle whereby the misbelief that secondary hypertension is exceptional translates into a very low rate of screening for secondary forms, thereby leading to identification of secondary hypertension only a small subset, thus reinforcing the misbelief that 95–99% of the patients have ‘essential’ hypertension.

In summary, the diffuse failure to search for the causes of hypertension has generated a vicious circle whereby the wrong belief that secondary hypertension is rare and, conversely, that essential hypertension is the most common form thus leading to a low rate of screening for secondary forms (Fig. 1). As discussed below, this disaster entails a multifaceted problem, which requires solutions, as the implementation of several actions, among which updating medical education to increase the awareness of the high prevalence of secondary hypertension, simplifying the screening and diagnostic work-up, and reassurance about the beneficial effects on outcomes.

### The prevalence of secondary hypertension

The prevalence of essential hypertension can be assessed by another perspective, namely by reviewing the studies that have attempted to determine how common secondary hypertension is. As mentioned before, the limitations described above for the studies on prevalence of essential hypertension also apply to studies on secondary hypertension, as most of the studies carried out thus far to ascertain the prevalence of secondary hypertension by no means fulfilled the criteria described in Table 1.

Reassuringly, there are, however, few exceptions, that entail mainly primary aldosteronism, which is now recognized as the most common form of secondary hypertension. In December 2006, the PAPY study for the first time used a predefined protocol and satisfied most of the criteria listed in Table 1. It prospectively recruited a large cohort (1125) of hypertensive patients consecutively referred to specialized hypertension centre nationwide in Italy and used a state-of-the-art diagnostic work-up and predefined cut-off values for the aldosterone-renin ratio [23]. It showed that 11.2% of the patients had primary aldosteronism, thus indicating that just one form of secondary hypertension has a prevalence two-fold higher than commonly estimated for all forms together in a study by Berglund *et al.*[11]. Importantly, half of the primary aldosteronism patients in the PAPY study had a unilateral form of primary aldosteronism that was surgically cured and the majority did not have hypokalaemia, the sign that commonly alerts doctors on the possibility, as already described by Dr Conn in 1965 [24], that primary aldosteronism can masquerade itself as essential hypertension.

Further large-scale surveys carried out thereafter confirmed the high prevalence of primary aldosteronism: 5.9% in an unselected population of hypertensive patients seen at the level of general practice [25], and 4% in China [26]. Even more important, a retrospective study performed in Greece suggested that the prevalence of primary aldosteronism can be much higher in patients with resistant hypertension [27]. This contention was eventually confirmed in the AVIS-2 Study, a large international study, which showed that resistant hypertension involves over 20% of the patients with primary aldosteronism and is a possible sign of presentation of primary aldosteronism [28]. In line with this, in a French randomized clinical trial on sympathetic renal denervation in patients with difficult-to-control hypertension, half of the 1416 patients initially screened, had to be excluded because they were found to have secondary hypertension [29]. This is an important piece of information because, patients with resistant hypertension can be investigated with adrenal vein sampling even while on multiple drug treatment and detected to have unilateral primary aldosteronism. In these patients, adrenalectomy provided striking benefits, both in term of resolution of high BP resistance to treatment and of regression of organ damage [30].

With regards to PPGL, the prevalence is not fully ascertained: general population studies in different countries reported a low prevalence and incidence, but all had limitations such as the retrospective design and use of public or national registries, or comprised meta-analyses of multiple small observational studies. A Danish study recently reported an age-standardized prevalence of 64.4 patients per million residents and an incidence of 6.6 per million person-years for PPGL [31]. A Dutch meta-analysis showed an incidence of 0.4 and 2.1 per million person-years for pheochromocytoma and paraganglioma, respectively. A parallel pathology study found a rate of 3.7 and 5.7 per million person-years. Similar figures of incidence were reported in a Canadian study, which showed an overall incidence of PPGL of 6.6 cases per million person-years [32]. However, it has to be acknowledged that 4.8% of the PPGL were found only at autopsy and never diagnosed during life, thus suggesting that real prevalence could be grossly underestimated due to missing clinical diagnoses [33].

Unfortunately, for other rarer forms of secondary hypertension, such as aortic coarctation, Cushing syndrome and renin-producing tumours, and even for other more common forms, including obstructive sleep apnoea, renovascular hypertension and hypothyroidism; currently, there are no studies fulfilling the criteria summarized in Table 1.

However, on the whole, even without considering other known causes of high BP, the data available for primary aldosteronism alone do not support the view that secondary hypertension is as rare as generally perceived and reported, but rather that it is quite common, albeit regularly overlooked.

Thus, the prevalence of secondary hypertension, albeit not generally known with precision for many forms, is much higher than usually held and the misbelief that secondary hypertension is exceptional is just due to the absence of systematic search of the causes of hypertension. Therefore, the afore-mentioned figures on the high prevalence of essential hypertension should no longer be presented as such in guidelines and textbooks.

These considerations underline the need to exploit strategies for the systematic search of secondary hypertension in clinical practice as already advocated, for example, for primary aldosteronism [34]. In our referral centre for hypertension, where this strategy has been deployed starting from the early 80 s, the proportion of identified cases of secondary hypertension has increased steadily and is now over 71%, thus indicating that essential hypertension is a vanishing entity.

### Essential hypertension is a life-sentence

Labelling a patient as affected by essential hypertension is not without serious consequences, because this is a chronic condition that, albeit treatable, is by definition incurable, since its cause(s) and underlying mechanisms are unknown. In the best scenario, following current guidelines [16], the patient diagnosed with essential hypertension will be instructed to implement lifestyle changes and will be prescribed antihypertensive medications, both of which measures should last life-long. Moreover, the need for antihypertensive medications usually increases over time, along with the development of organ damage. More often, the patient will only receive drug prescription which, while controlling the high BP values only in a proportion of the cases, will not eliminate the excess cardiovascular risk associated with persistently high BP in many patients, leaving them with a ‘residual risk’. Over time, several patients requiring an escalating dose of a single agent and/or more antihypertensive agents will experience side effects with the treatment. These two facts are known to negatively impact on patient's adherence to long-term treatment and contribute, along with other factors, to the disappointing control rate of high BP worldwide [35].

On the contrary, identification of secondary hypertension, and institution of treatment targeting the underlying cause, will permit to achieve cure of high BP and/or a decreased need for medications in most of the cases. Therefore, the arguments to encourage doctors to undertake all the efforts that are necessary to identify a curable cause of hypertension are strong.

### A hypertensionologist's dream

**FIGURE 2:**
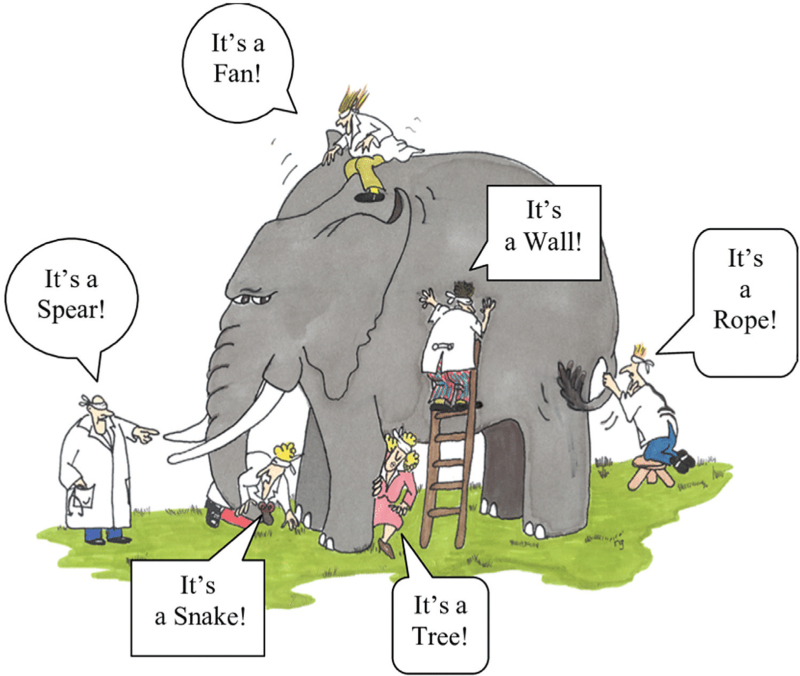
When facing a patient with essential hypertension without ascertaining the underlying pathophysiology, doctors are like the six blind-folded researchers examining the elephant in the cartoon: their understanding of real facts based on limited data is incomplete and, hence, inaccurate. Picture from: Himmelfarb *et al.* 2002 [48]: 1526 (artist: G. Renee Guzlas). All rights reserved ©.

The availability of a test, or a combination of tests, that could allow to accurately diagnose essential hypertension has been a hope for hypertensionologists for decades, because it could save an enormous amount of time and energy spent for identifying secondary forms of hypertension. Hence, in the quest for biochemical markers and genes and/or genetic mutations that could allow detection of essential hypertension, considerable efforts and investments of time and resources have been deployed. These efforts have unfortunately been rewarded by limited success, mainly in the field of endocrine hypertension, as discussed elsewhere [36]. Of note, a recent E.U.-funded international effort aimed at exploring the hypothesis that a multiomics-based strategy coupled to machine learning could allow a better detection of endocrine hypertension namely primary aldosteronism, PPGL and Cushing syndrome, has been rewarded by some success in that it allowed to identify some signatures of these conditions, but not of essential hypertension [37,38], which remains an ‘exclusion’ diagnosis. Therefore, when facing a patient with essential hypertension without ascertaining the underlying pathophysiology, doctors are like the six blind-folded researchers examining the elephant in the cartoon: their understanding of real facts based on limited data is incomplete and, hence, inaccurate (Fig. 2).

At present, we would like to contend that good clinical practice should entail a systematic search of all the causes of arterial hypertension, which can be achieved by applying the simplified diagnostic algorithms that are available at least for the most common forms of secondary hypertension, as discussed elsewhere [19,39–42]. This search is particularly important in some categories of patients, in whom the benefits are much rewarding. These include pregnant women [43,44], patients who are young and/or have stage II-III hypertension, those with resistant hypertension, who have a high probability of having secondary hypertension and in whom, by definition, medical treatment fails, thus putting then at the highest risk of all [30].

## CONCLUSION AND PERSPECTIVES

Thus far, investigative efforts and hypertension guidelines, with the notable exception of the 2017 AHA Hypertension Guidelines, have devoted most attention to phenotypic characterization, that is to the measurement of BP and confirming the diagnosis of hypertension, and to provide recommendation or suggestions for drug treatment [14]. Quite weird, in a time when the hot topic in Medicine is a precision approach, little focus has been put on the search for ascertaining the patophysiology and the causes of human arterial hypertension. Therefore, the time has come, in the field of clinical research in arterial hypertension, to move from a phenotypic/observational approach to mechanistic investigation. From the research standpoint, this means moving resources towards investigation to identify novel molecular targets, with the goal of developing strategies for a tailored individual management approach with use of mRNA technology, as recently done [45]. This could eventually allow long-term cure of high BP, better outcome for patients and substantial savings of the financial resources that are currently devoted to life-long pharmacological treatment of hypertension. From the practical standpoint, this implies undertaking a systematic search for the cause(s) of hypertension and, therefore, identification of the underlying pathophysiologic mechanisms. Discovering secondary hypertension is the premise for instituting a targeted treatment, which is usually more effective and, in several instances, curative as shown for primary aldosteronism [46].

## ACKNOWLEDGEMENTS

### Conflicts of interest

There are no conflicts of interest.
